# Vestibular activity and cognitive development in children: perspectives

**DOI:** 10.3389/fnint.2013.00092

**Published:** 2013-12-11

**Authors:** Sylvette R. Wiener-Vacher, Derek A. Hamilton, Sidney I. Wiener

**Affiliations:** ^1^Vestibular and Oculomotor Evaluation Unit, Department of Otorhinolaryngology, Robert Debré Pediatric HospitalParis, France; ^2^Department of Psychology, University of New MexicoAlbuquerque, NM, USA; ^3^Laboratoire de Physiologie de la Perception et de l’Action, UMR-7152, Centre National de la Recherche Scientifique - Collège de FranceParis, France; ^4^Memolife Laboratory of Excellence, Paris Science and Letters UniversityParis, France

**Keywords:** ontogeny, development, vestibular, otolith, cognitive, navigation, human

## Abstract

Vestibular signals play an essential role in oculomotor and static and dynamic posturomotor functions. Increasing attention is now focusing on their impact on spatial and non-spatial cognitive functions. Movements of the head in space evoke vestibular signals that make important contributions during the development of brain representations of body parts relative to one another as well as representations of body orientation and position within the environment. A central nervous system pathway relays signals from the vestibular nuclei to the hippocampal system where this input is indispensable for neuronal responses selective for the position and orientation of the head in space. One aspect of the hippocampal systems’ processing to create episodic and contextual memories is its role in spatial orientation and navigation behaviors that require processing of relations between background cues. These are also impaired in adult patients with vestibular deficits. However little is known about the impact of vestibular loss on cognitive development in children. This is investigated here with a particular emphasis upon the hypothetical mechanisms and potential impact of vestibular loss at critical ages on the development of respective spatial and non-spatial cognitive processes and their brain substrates.

## INTRODUCTION

How does loss of vestibular function at various ages of childhood impact on the development of complex spatial behaviors and cognition? To respond to this, it is necessary to chart the ontogeny of these behaviors and of the brain structures implicated in their expression. Bilateral loss of vestibular function at or close to birth results in motor developmental delays (Rine et al., 2000; Whitney et al., 2009; Wiener-Vacher et al., 2012b). Although vestibular loss can be compensated with a return to normal postural and oculomotor functions, observations of such children throughout childhood reveal that many of those with complete vestibular loss exhibit learning disabilities and poorly adapted strategies for overcoming their sensory deficit (Franco and Panhoca, 2008). For example, the gaze and fixation problems associated with vestibular dysfunction can lead to reading problems requiring specific therapy (Braswell and Rine, 2006). Development of diverse cognitive functions could be impaired in vestibular-deficient children through several possible mechanisms. For example, vestibular deficits can impair detecting and distinguishing one’s own movements from other movements in the environment through both the visual and proprioceptive systems.

It has also been hypothesized, similar to the “critical periods” observed for visual system development, that other cognitive functions also have limited developmental windows when their underlying brain structures establish long-lasting connectivity with repercussions for life. During movements, sensorimotor loops transmit conflicting or inaccurate information in vestibular-impaired patients and this could lead to faulty wiring and deficits in cognitive function. This chain of events can be conceptualized in a framework where high level brain representations are built up from sensorimotor loop activity by intermediate representations of emulated or imagined actions in the real world and their anticipated outcomes.

Vestibular patients have difficulties in constructing and using several types of brain representations of space. Adults with bilateral vestibular lesions have hippocampal atrophy and suffer spatial and non-spatial cognitive impairments (Schautzer et al., 2003; Brandt et al., 2005). Do critical periods exist during development where vestibular signals are required to establish normal hippocampal circuitry permitting spatial navigation and other functions? To address this issue, theoretical background will first be provided on the forms of spatial navigation, noting how they are supported by the diverse types of vestibular information. Next we will discuss the hippocampal system, its spatial representations, its relations with the vestibular system and its development, followed by a review of developmental studies of performance in specially designed spatial orientation tasks. The final section will tie this all together, leading to specific predictions of the impact of vestibular damage on spatial cognition at respective ages corresponding to milestones in brain and cognitive development. This will be considered in terms of early identification of the potential cognitive deficits deriving from vestibular disorders, thus permitting better adapted therapy and training programs.

Vestibular inputs provide several types of information which are respectively engaged for diverse corresponding sensorimotor and cognitive processes (Potegal, 1982; Wiener and Berthoz, 1993; Borel et al., 2008). The three pairs of semicircular canals and the otolith organs provide complementary information about several types of rotational and translational head movements involving accelerations and, importantly, signal the direction of gravitational force. The latter is fundamental to terrestrial life from birth since postural adjustments and active movements invariably must take gravity into account. The brain builds representations of verticality based upon vestibular, somatosensory proprioceptive, and visual information, constructing a “geocentric” reference frame (Borel et al., 2008). Vestibular patients are thus handicapped in acquiring information during active displacements in the environment since sensory frames of reference (e.g., visual or proprioceptive; Lacour et al., 1997; Isableu et al., 2010) must be established without vestibular information. Furthermore, gravitational and other vestibular information can be instrumental in timely acquisition of fundamental spatial relationships of up vs. down, left vs. right, front vs. back, etc. (Wiener-Vacher et al., 2012a). Infants first learn these spatial relations with reference to their own body. Understanding spatial relations between the body parts is difficult for vestibularly impaired infants, perhaps in part because proprioceptive information about gravity is not reinforced by otolithic gravity sensation. This would then have a negative impact on understanding other spatial relationships such as over/under, inside/outside, within/without, interposition, etc. These patients would then have difficulty applying these concepts for establishing coordinate systems for representing the relative positions and orientations between environmental features and their own relative position and orientation to all of this. Furthermore, if concepts like “close, distant, superior, inferior, etc.” are poorly understood, the child could also have difficulty extending them to arithmetic and geometry as well as to other non-spatial domains where sets and groups are compared (e.g., syntax, biology, history).

The vestibular system also makes a vital contribution in helping to distinguish visually perceived self-movements from movements of objects in the environment. Vestibular inputs help to reconcile diverse and conflicting signals including vision, proprioception (and other somatic sensation) and internally generated motor commands. For example, *optic flow* signals are generated when the head moves slowly at a constant velocity, but they also occur when the head is immobile by viewing clouds drifting across the sky, movement of the environment as seen from a stroller or a car window, by points of light projected by a rotating disco mirror ball, by movements of crowds, while seated on an immobile train when the train on the next track pulls away, etc. Difficulties in reconciling self-movements from non-self-movements as well as in selecting appropriate vertical and horizontal references can thus lead to problems in postural and motor coordination, fine motor control, and visual processing. Vestibular patients depend more on vision and proprioception for determining the earth vertical orientation and if an object taken to be a stable reference point moves, this can lead to postural instability and disorientation.

Signals related to rotational and linear accelerations including gravity can help stabilize and inform several types of movements. Each of these is associated with cognitive processes that can lead to distinctive types of problems in cases of vestibular impairments. These movement types include:

(a) gaze stabilization during passive and active head and body movements,
(b) maintaining equilibrium: stable head on neck and body posture during immobility and movements, both passive and active,
(c) relative movements among body parts (head on neck, pointing, touching parts of head and body),
(d) locomotion,
(e) interaction with the environment (pushing, reaching, catching, ducking/avoidance, etc.).

Impairment or late development of these functions would also deprive the patient of the sensorimotor feedback information generated by these movements. For example, infants without otolith function learn to walk later than controls (Wiener-Vacher et al., 2012b) and fall more frequently. This developmental deficit means that they do not receive timely and coordinated visual and proprioceptive feedback associated with stable walking – information that would be vital for building spatial representations. An infant with a vestibular deficit, who typically walks very cautiously and attentively, seeking mechanical support and maintaining a rigid neck, is not able to learn as much about spatial relationships in the environment, and thus will have less opportunity to build internal representations of space. For example, distances are often calibrated in numbers of paces, but this is not feasible for these patients. One theoretical framework of how cognitive representations emerge in the brain contends that sensorimotor loop activity is internally simulated and re-represented in the absence of the relevant sensory inputs and movement. This would lead to anticipatory processes and the construction of yet higher level representations. Since vestibular dysfunction would impair the many sensorimotor processes described in the previous paragraphs, serious consequences can be expected in building representations and cognitive processing in the corresponding functional domains.

Before reviewing the literature relevant to the question of the developmental consequences of vestibular impairments, it is necessary to re-emphasize that the semicircular canals and otolith organs respectively provide fundamentally different information. In particular, only the otoliths are specialized for detecting the direction of gravity force crucial for establishing vertical orientation and thus defining spatial reference frames in concert with the axes of the rotational selectivity of the semicircular canals. In the vast majority of the literature, the *patients groups described as “vestibular impaired” were tested for semicircular canal function* only. *Thus it is possible that residual otolith organ function remained in some reportedly vestibular-deficient patients – and that some reportedly normal controls had functional canals but no otolithic responses*. Even for experimental subjects who have had surgical labyrinthectomies or neurectomies, it is advisable to perform comprehensive vestibular testing to verify that there is no residual function. A second issue is that patients show a great deal of variability in their degree of compensation due to unequal access to adapted training or therapeutic life experiences. It is possible that individuals may differ in central compensation processes – evidence has been found for increases in volume in bilateral connections between the vestibular nuclei, in proprioceptive processing area of right gracile nucleus and the visual motion processing area MT/V5 (zu Eulenburg et al., 2010). Furthermore some may simply learn more effectively to substitute other cues such as visual field flow, various types of proprioceptive cues, visceral enteroception, visual landmark cues, and various vertical/horizontal cues. These caveats should be recalled in interpreting results from the literature and in planning new experiments.

## TYPES OF NAVIGATION PROCESSING

Orienting cues can be considered in two categories by virtue of whether they transmit information about self-motion or about environmental characteristics. Self-motion cues come from the vestibular system, enteroceptors (located in the abdomen), motor efferent collaterals related to locomotion and orienting movements, proprioception countering gravitational forces and also transmitting traction or slippage of paws or feet against the substrate during locomotion. Although the vestibular system is only sensitive to rotational or linear acceleration of the head, the brain mathematically integrates these inputs over time first to provide velocity signals, and then again to detect the angle rotated and the linear distance covered. These integrations are subject to drift errors and are generally not reliable for displacements lasting longer than about 10 s, requiring regular corrective updates, for example, by consulting with visual cues. Studies of animals passively displaced then required to return to their nest show that rotations are taken in account more than translations (Etienne et al., 1988). Another important self-motion signal comes from optic field flow. This is the coherent movement of the image of the entire visual field relative to the eyes during head movements and it indicates the velocity of the head in space. While optic flow derives from visual detection of environmental cues, it cannot be accurately described as “allocentric.” In vestibular rehabilitation therapy, patients learn to substitute the various visual and proprioceptive cues described above. Finally, information about the environmental layout comes chiefly from visual perceptions of objects, audition, and in certain species, magnetic sense, echolocation, and other exotic senses.

Diverse types of navigation strategies engage distinct cognitive processes (Trullier et al., 1997). Firstly, in *dead reckoning*, or *path integration*, the initial starting point is noted. Then while traveling, the velocity is integrated over time to compute the distances covered. Angular heading during these displacements is taken into account by vectorial addition yielding the total displacement as a result (and reversing this gives the return vector). Principal sources of information are the self-motion cues described above, including the vestibular sense. Correct estimation of the duration of time is clearly crucial for this integration (Israël etal., 2004).

The *body alignment and target approaching* navigation strategy, also referred to as *beacon homing*, *piloting,* or *approach/avoidance*, involves moving toward (or away from) a cue or object in the environment. In the *guidance* strategy (as defined by O’Keefe and Nadel, 1978), the animal maintains a certain egocentric relationship with respect to a particular landmark or object. A vestibular patient with oscillopsia (continual oscillation of the visual field) might be expected to have difficulty with this.

The next categories of navigation strategy are more advanced since they can be used to reach a known but not currently visible goal and involve identification of and orienting relative to *places*. A place is defined within a large-scale environment as a set of contiguous locations that are equivalent with regard to action selection (Trullier et al., 1997). A place can also be defined as the set of locations from which a set of landmarks or a landmark configuration is perceived as identical or very similar. Thus implicit to this is a capacity to make generalizations. The term *place navigation* refers here to navigation toward a specific location based on its spatial relationship to a constellation of exteroceptive cues, particularly distant background visual cues. Other strategies such as piloting or vector-based navigation (Pearce et al., 1998) can be distinguished from place navigation in that no single cue is sufficient for place navigation.

Returning to the types of navigation processes, in *place recognition-triggered responses*, the origin and intermediate places along the route each have an associated angle of departure and distance to go to the next place. *Topological navigation* involves three steps: (a) recognizing the place where one is currently situated; (b) orienting within this place; and (c) selecting in which direction to move so as to reach its current goal. It is not necessary to plan a sequence of subsequent movements, but only to select the very next action. *Metric navigation* implies a veridical internal map that is consulted for making the most efficient changes in position.

## VESTIBULAR DYSFUNCTION AND DEFICITS IN COGNITIVE FUNCTIONS INCLUDING NAVIGATION

Vision is a primary sensory modality in humans for detecting size, shape, distance, and layout information. Static and dynamic visual acuities are impaired by vestibular deficits. The ability to maintain a stable visual image while the head is moving, such as during walking, is dependent upon visual and vestibular inputs triggering eye movements opposing and compensating for head movements. When vestibular function is normal, visual acuity is similar whether the head is moving or stationary. The difference in static and dynamic visual acuity (DVA) can be quantified using the DVA test (Schubert et al., 2006). Adults and children with vestibular deficits have impaired DVA (Rine and Braswell, 2003; Herdman et al., 2007). Vestibular deficits are characterized by an absence of the vestibulo-ocular response which maintains gaze fixed on a target when the head is passively moved suddenly in the direction of sensitivity of a vestibular receptor end organ. This is the basis of the commonly used clinical head impulse test (HIT) for detecting vestibular deficits (Halmagyi et al., 1994). Subjects with complete vestibular loss complain of oscillopsia during movements – this makes them dizzy and disoriented when they walk, run, drive, and read. Indeed, Braswell and Rine (2006) reported that children with vestibular deficits have poor DVA results and this is associated with significant reduction in reading acuity. Smooth pursuit oculomotor activity can compensate for head rotations up to a velocity of 100°/s. However, above this speed only the vestibular system can detect and compensate for movements, and this range of sensitivity is needed for many activities of daily life. Indeed, walking induces much low amplitude but high acceleration and velocity vibration and shaking of the head (as apparent in a movie taken from a camera carried on one’s shoulder) and the vestibular system permits this to be transformed to a smooth continuum.

In addition to problems related to vision, vestibular deficits can lead directly to difficulties in estimating angular and distance displacement, presumably through path integration. Beritoff (1965) observed navigation deficits in children and in experimental animals with no detectable semicircular canal function. In cases where the animals were familiar with a trajectory to a reward site, in the absence of vision, they no longer were able to go directly to the learned reward site. When vision was restored, they resumed taking the direct path. Children aged 10–12 years old were blindfolded, led or carried along a trajectory then along the return path. They were able to retrace the steps, while blindfolded children with non-functioning labyrinths could not, even after several trials. This is one of the rare studies examining cognitive deficits in vestibular-impaired children; the following text examines the literature concerning adults. These studies show that vestibular patients have difficulties in detecting and estimating body displacements in the dark. During goal-directed locomotion, these patients make errors in trajectory (e.g., Borel et al., 2004; Cohen and Sangi-Haghpeykar, 2011). Another test where they have difficulty is reversing the trajectory along a triangular path or finding a shortcut (Péruch et al., 1999,^2005^; Glasauer et al., 2002; Guidetti et al., 2007; for review, see Israël etal., 2005).

 In experiments evaluating dead reckoning, rats were required to make return trips to a hidden start location under dark conditions (Wallace and Whishaw, 2003). The peak velocity was observed at the point midway of this return trajectory and the direction of this trajectory was highly accurate, suggesting the computation of both the distance and direction to return to a target point of origin, consistent with dead reckoning. In similar experiments this team also demonstrated that dead reckoning is impaired after chemical labyrinthectomy (Wallace et al., 2002).

A great deal of contemporary understanding of place navigation and its neurobiological bases has come from research using the Morris water maze task (Morris, 1981,^1984^; Sutherland and Dyck, 1984). In this task, rodents (typically rats) learn to navigate to an escape platform submerged in a circular pool of cool, opaque water. Because the circular pool provides only information about radial distance from the border, animals learn to navigate to the escape platform by way of reference to a constellation of visual cues outside the pool.

Over the past 15 years, several laboratories have utilized computerized, virtual, navigation tasks based on the Morris water task to measure place navigation in humans (Astur et al., 1998; Jacobs et al., 1998; Hamilton and Sutherland, 1999; Doeller and Burgess, 2008; Doeller et al., 2008; Mueller et al., 2008; Hamilton et al., 2009). The participants view an environment from a first-person perspective and “swim” in the virtual space using a keyboard or joystick. As in the Morris water maze, the environment contains distal visual cues and the subject must navigate to a hidden goal. These tasks have been shown to both engage (Cornwell et al., 2008; Doeller and Burgess, 2008) and require an intact hippocampus (Astur et al., 2002; Driscoll et al., 2003; Hanlon et al., 2006). The computerized virtual Morris water task (VMWT) has been used to characterize spatial memory deficits in patients with developmental disturbances (e.g., fetal alcohol syndrome, Hamilton et al., 2003) and psychiatric disorders (e.g., schizophrenia, Hanlon et al., 2006). Hartley et al. (2003) found fMRI activation in the hippocampus of human subjects during a virtual wayfinding task. Human subjects performing a virtual task requiring them to point to the origin of a trajectory along two sides of a triangular path also show increased activation of the hippocampus (Wolbers et al., 2007). Caloric vestibular stimulation activates the hippocampus in humans (Vitte et al., 1996). Although the head is fixed and thus there are no vestibular signals that are generated or required for this task, the relationship between vestibular function and performance has been examined in several studies (Schautzer et al., 2003; Brandt et al., 2005; Hufner et al., 2007). Patients with bilateral vestibular failure are impaired at finding the hidden platform, but perform as well as matched controls in navigating to the platform when it is visible. Navigation deficits were far more subtle in unilateral vestibular-deficient patients, and only appeared in patients with right, but not left, vestibular failure (Hufner et al., 2007). Structural analyses via magnetic resonance imaging revealed that hippocampal volumes were significantly decreased in bilateral vestibular patients (Brandt et al., 2005), whereas major volumetric reductions in unilateral patients were limited to gray matter reductions in the cerebellum, temporal neocortex, thalamus, and area MT/V5 (Hufner et al., 2009).

Vestibular patients are also impaired in object-based mental transformations, another example of a cognitive task performed with the head immobile and thus in the absence of self-movement cues that would engage the vestibular system (Péruch et al., 2011) The experimental groups were Menière’s patients after unilateral vestibular neurectomy, patients with bilateral vestibular damage and normals. One task required mental rotation of 3D-objects and two other tasks involved mental scanning and tested the ability to construct and manipulate mental images with metric properties. The authors reported variations in performance corresponding to the level of vestibular loss. Bilateral vestibular patients often had the worst results. The Menière’s patients showed greater deficits early after neurectomy and then gradually compensated. This is of particular interest because it demonstrates a role for vestibular signals in processing metric properties of mental representations, supporting the hypothesis that high level processing is in play.

It is fairly common for vestibular patients to have difficulty detecting and estimating the magnitude of passive body displacements in the dark. During goal-directed locomotion, these patients usually make errors in executing the desired trajectory (e.g., Borel et al., 2004; Brandt et al., 2005; Cohen and Sangi-Haghpeykar, 2011). Spatial disorientation is even stronger during complex tasks such as reversing the trajectory along a triangular path or finding a shortcut (Péruch et al., 1999,^2005^; Glasauer et al., 2002; Guidetti et al., 2007). Péruch et al. (1999) found that unilateral vestibular loss impairs the orientation component (estimation of the angular displacements) of navigation. The distance component (estimation of the linear displacements) of the spatial representation is also impaired, although to a lesser extent.****

Self-motion or optic field flow in the opposite direction can elicit comparable perceptual, motor, and neurophysiological responses. Convergence of visual field flow and vestibular inputs have been observed in many structures including the vestibular nuclei (Xerri et al., 1988), vestibular area 2v (Büttner and Buettner, 1978) and the parieto-insular vestibular cortex (Grüsser et al., 1990).

Hanes and McCollum (2006) identified cognitive deficits associated with vestibular dysfunction including short-term memory, concentration, arithmetic, and reading. For example, patients with central vestibular lesions required to count backward by twos make more errors and are slower than controls. This was interpreted as resulting from “spatialization” of the task, perhaps in terms of number line representation. Performance impairments can be categorized as direct, that is, tasks that implicitly or explicitly require using information about the 3D structure of space and movements (such as navigation and spatial memory). This also includes the use of spatial strategies in non-spatial domains. Of particular interest here is that a common strategy of skilled mnemonists is to employ mental imagery of places and signs to situate information to be memorized. It would then follow that spatial cognitive deficits could limit the capacities of patients for this type of memorization skill.

Indirect effects of vestibular deficits on cognition derive from the greater demand on attentional and cognitive processing resources at the expense of other ongoing activities (Smith et al., 2005b). For example, the lack of vestibular information requires the sometimes effortful substitution of visual, proprioceptive, and other signals in order to maintain balance, posture, and gaze. Visuospatial processing is also more difficult. This reduces attention, limits concentration and could tie up mental processing resources, impairing other activities such as multi-tasking, processing sequences, and attention-shifting. Patients could thus have difficulty organizing multiple sources of information, in particular integrating new information while retaining previous items in memory – this could impair problem solving and conflict resolution. All of these are important for spatial orientation and navigation. For example, routes are often schematized in terms of sequences of intermediate goals and the associated trajectories to be followed to the next intermediate goal.

Note also that vestibular deficits often report sensations such as vertigo, disorientation, discomfort with repeated peripheral patterns during movement, etc. These then are associated with psychiatric problems such as agoraphobia (such environments provide troubling cue conflicts), excessive fatigue, depression, and anxiety. Overall, these all can lead to indirect negative impacts on measures of spatial and non-spatial cognitive processing.

## PROCESSING OF VESTIBULAR SIGNALS FOR SPATIAL REPRESENTATIONS IN THE HIPPOCAMPAL SYSTEM

Head direction (HD) cells fire when the head of the rat (or mouse, or chinchilla) is oriented in a particular direction in the yaw plane, regardless of its position in the environment (Ranck, 1986; Taube et al., 1990; Muir et al., 2009; Yoder and Taube, 2009). HD responses are found in all of the brain areas designated as the Papez circuit, running from the brainstem to the hippocampus. The signals are generated in the brainstem lateral mammillary nucleus and dorsal tegmental nucleus (DTN) of Guddens which receives inputs from the vestibular nuclei (Bassett and Taube, 2005). Although the direction responses are anchored by background visual cues (likely distinguished by motion parallax; Zugaro et al., 2001) and are influenced by optic flow stimuli (Arleo et al., 2013), they remain selective for direction in darkness (e.g., Yoder and Taube, 2009). Stackman and Taube (1997) injected sodium arsanilate in the vestibular end-organs of rats, and this abolished the directional responses in the anterodorsal thalamus. Yoder and Taube (2009) studied HD cells in a mouse strain with nearly complete absence of otoconia and hence minimal otolith function. HD cells were observed but signals were more weakly controlled by visual landmark cues, and responses degraded over successive trials and were unstable in darkness.

Principal neurons of the hippocampus discharge selectively as the rat, mouse, or human occupies a particular position in its real or virtual environment (O’Keefe and Dostrovsky, 1971; Ekstrom et al., 2003; Chen et al., 2013). This activity is considered to participate in an internal representation of the environment (O’Keefe and Nadel, 1978). Indeed, during immobile pauses prior to locomotion, these cells fire in rapid sequences corresponding to the imminent trajectory the rat is about to take (Pfeiffer and Foster, 2013). Vestibular lesions suppress these place responses (Stackman et al., 2002; Russell et al., 2003a) and cause other changes in hippocampal physiology (Smith et al., 2005a; Russell et al., 2006). Furthermore, rats with hippocampal lesions are impaired in orienting to a goal after being passively rotated (Mathews et al., 1989) and in spatial learning (Russell et al., 2003b).

Place learning in the Morris water task critically depends upon intact circuitry upstream of the vestibular organs leading to hippocampus (and involved in the generation of HD cell signals; Vann et al., 2003; Clark and Taube, 2009; Clark et al., 2013) as well as the hippocampus itself and related structures (Morris et al., 1982; Sutherland et al., 1982). These patterns of damage can leave other forms of navigation, such as cued navigation, unimpaired.

Comparisons of place responses in hippocampal neurons of rats before and after rotation of the experimental arena in darkness revealed that a subset of neurons maintained their firing fields at the same position in absolute space, rather than rotating with the apparatus (Wiener et al., 1995). This was interpreted to indicate that the brain had detected the angle of rotation, perhaps via the horizontal semicircular canals, then compensated for it by stabilizing the hippocampal position representation. Since proprioceptive cues may have also played a role in this, a new experiment was devised where the head of the rat was immobilized, its body suspended in a hammock (with the leg protruding through holes), and passively displaced on a mobile robot (Gavrilov et al., 1998). Hippocampal place responses were recorded under light conditions, and they persisted in complete darkness. This provided further, and more direct support for vestibular updating of hippocampal spatial representations. In this same experimental protocol, passive rotations in the dark synchronized hippocampal local field potentials to rhythmically oscillate at 8 Hz, the “theta” rhythm, which is associated with locomotion and active exploration (Gavrilov et al., 1996).

During locomotion in an open field, hippocampal responses in a given place are the same regardless of the orientation of the head, and hence the view perceived by the rat, which is a form of abstraction (Wiener, 1996). This suggests that there is a memory process associating the successive multiple views to produce the same cellular response, presumably reflecting a single coherent representation. One way for the brain to detect that the head is in a certain place would be to compute the distances and angular headings of at least two environmental landmarks. This would require simultaneous storage and comparison of this information, implicating working memory and multi-tasking, processes associated with the hippocampal–prefrontal cortex pathway. Since vestibular lesions abolish place cell activity and induce hippocampal atrophy, perhaps these losses could also impair these processes as well as affecting memory in spatial and non-spatial domains as observed after hippocampal lesions.

Grid cells of the entorhinal cortex (situated in the pathway from the HD system to the hippocampus) discharge as a rat occupies places that are distributed along the nodes of a hexagonal grid within its environment (Moser et al., 2008). Thus these neurons provide a coordinate reference frame for navigation. No study has yet tested the effects of vestibular lesions on grid cells. However, computational models of grid cells require head orientation input – and HD cells are also found in entorhinal cortex. This, and the additional computational requirement for self-displacement signals, indicates that vestibular signals would also be required for grid cell activity.

If vestibular-deficient patients do not have place, HD or grid responses in their hippocampal system, this would deprive them of valuable spatial signal processing and representation capacities. Furthermore, the absence of these signals during development could impair the construction of circuits underlying orientation and navigation behaviors, and perhaps other cognitive functions that these areas contribute to as well. Indeed, eventual hippocampus mis-wiring in the absence of vestibular inputs could also have an impact on non-spatial cognitive processing (Wiener, 1996) by this structure as well and on signaling to downstream structures like prefrontal cortex and ventral striatum.

## DEVELOPMENT OF BRAIN REPRESENTATIONS OF THE ENVIRONMENT AND ORIENTATION CAPACITIES IN RATS

During the first few weeks of postnatal life the navigational capacities of the rat and other rodents develops rapidly. Rat pups first venture out of the nest around postnatal days (PD) 10–11 (Bolles and Woods, 1964) and rapidly increase explorations there around PD 16–19 (Alberts and Leimbach, 1980). (Rat pups first open their eyes on PD 15, the same age that they start to walk while bearing the body weight). These exploratory trips appear to be directed not only by internal motivational cues and biologically significant proximal cues (e.g., heat sources) but also to acquire information about distal visual cues (Loewen et al., 2005). During this period the circuitry of the hippocampus and related structures also undergo significant structural and functional development (Bachevalier and Beauregard, 1993; Dumas, 2005). It is generally believed that maturation of the hippocampus is delayed compared to other brain regions, rendering rats incapable of performing hippocampal dependent tasks until at least PD 19–25 (Bachevalier and Beauregard, 1993; Stanton, 2000; Dumas, 2005). A growing body of data from studies investigating the ability of young rats to navigate, however, suggests that the neural systems involved in navigation may be functional even earlier than this. Of particular interest are studies examining the ontogeny of spatial firing characteristics of neurons in the hippocampus and related brain regions implicated in spatial navigation and memory. Langston et al. (2010) reported that the activity of HD cells in the pre- or parasubiculum of preweanling rats displayed adult-like properties at PD 15–16 and the proportion of HD cells was similar to that of the adult animal. Although hippocampal place cells displayed spatially selective firing and medial entorhinal grid cells displayed their characteristic spatially periodic firing shortly thereafter (PD 16–18), the spatial firing patterns of these cells either continued to become more precise and mature and the proportion of responsive cells continued to increase toward adult levels over the next 10–17 days (Langston et al., 2010; but see Wills et al., 2010,^2012^). Overall, these observations suggest a primacy of directional processing by HD cells, which is followed by the maturation of place and grid cell signals, respectively (Ainge and Langston, 2012). If hippocampal place cells and the directional tuning observed in some grid cells depend upon HD cells (Knierim and Hamilton, 2011), it is perhaps not surprising that HD cells also mature earlier. These considerations would lead to the expectation that behaviors guided by orientation signals provided HD cells should emerge earlier in development than more complicated cognitive functions such as place navigation (Ainge and Langston, 2012).

Akers et al. (2011) adapted the Morris water task in order to develop a more sensitive assessment of control of navigation by distal visual cues. Prior work from this group examined the effects of translating the pool to another overlapping position in the room with salient visual cues on the walls (i.e., shifting it within the distal cue reference frame). The rats were first trained to navigate to a hidden platform, the pool was displaced in the room, and the rats could swim either to the same precise location in the room where the platform was previously located or to navigate toward the previous location relative to the pool border, respecting its orientation relative to the room cues (Hamilton et al., 2007,^2008^,^2009^). The rats chose the latter, suggesting that the distal cues can be engaged for orientation information while precise spatial location is based on the local frame of reference (the pool border). The possible outcomes of the translation test described above were recently dissociated by Stackman et al. (2012) in the mouse. Following training these authors pharmacologically inactivated either the anterodorsal thalamus or CA1 subfield of the hippocampus prior to the translation test. Mice with CA1 inactivation navigated to the relative location relative to the pool, whereas mice with thalamic inactivation preferred the location in the room, supporting the contention that navigation based on orientation relative to distal room cues depends on thalamic HD cells.

Hamilton et al. (2007) also trained rats to navigate to a cued platform (i.e., marked by a conspicuous visual cues) in the same distal room environment as used in the hidden platform task. After the rats mastered performance the pool was translated while the cued platform either remained in the same location relative to the room cues or the same location relative to the pool border. The rats succeeded at the latter, but surprisingly, when the cued platform was placed in the same precise location relative to the room cues, but in a different part of the pool, the rats first navigated in the direction of the platform’s previous position relative to the pool walls (respecting the orientation of the room) before correcting course to the cued platform. Thus they initially ignored the cues co-localized with the platform, and instead relied on the pool border in relation to the room orientation, suggesting a priority for this type of navigation strategy at the expense of beacon utilization. These observations provide further evidence that distal cues can control orientation independently of processes that would determine precise spatial localization, and are consistent with previous work by the same group showing that the initial orientation of swim trajectories to a cued platform in the water task are controlled by distal room cues, whereas the proximal cue co-localized with the platform guided subsequent navigation (Hamilton et al., 2004). Interestingly, this dissociation was hinted at by the fact that rats tended to engage in head-scanning behavior after navigating a short distance from the release point. Further manipulations such as changing room cues or relocating the cued platform revealed that these head-scanning behaviors marked the transition between control by distal room cues and control by the proximal cue.

Recently, Clark et al. (2013) demonstrated that lesions of the DTN (part of the brainstem circuit processing vestibular signals to generate HD cell activity) dramatically impair the engagement of distal cues in this Morris water task. Using the cued navigation task described above these authors demonstrated that rats with DTN lesions directly to the cued platform regardless of its position in the room and pool during the translation test. Akers et al. (2011) also utilized this variant of the task to examine the developmental trajectory of orientation control by distal cues. Interestingly, rats at PD 16 showed no significant difference in latencies to the cued platform whether it was in the same location in the pool or room, whereas all rats PD 17 or older displayed the adult pattern of outcomes, first erroneously swimming to the previous position relative to the pool walls, as guided by its orientation relative to the room. Most studies indicate that the emergence of place navigation in rats begins between PD 20–22 (review: Akers and Hamilton, 2007) which is generally taken to reflect the maturation of the hippocampus, supported by upstream sensory and cognitive systems involved in navigation. The observations of Akers et al. (2011) are consistent with the hypothesis that distal cues control orientation, but not precise position, very early in development, at the same time that HD cells are maturing functionally, prior to the appearance of mature place cell and grid cell responses.

## HIPPOCAMPAL DEVELOPMENT IN CHILDREN

Knickmeyer et al. (2008) reported a 13% increase of hippocampal volume from the ages of 1 to 2 years (but relatively little growth could be seen after it was normalized for total brain volume). Giedd et al. (1996) found that right hippocampus growth (normalized with respect to cerebral volume) correlated with age only in females, and that the left hippocampus did not increase with age between 4 to 18 years in males, or females. Uematsu et al. (2012) employed a cubic regression to chart the developmental trajectories of hippocampal regions. Their data show increases in hippocampal volume during the first 6–7 years of life, with a peak at about the age of 10–11. Gogtay et al. (2006) performed a volumetric study of MRI scans from humans aged from 4 to 25. They observed that total hippocampal volume does not change over this period although there are regional variations. Concerning connectivity, Ábrahám et al. (2010) showed that myelination progresses differently in hippocampal subregions, reaching adult levels in fimbria-fornix, stratum lacunosum-moleculare and alveus at 3 years of age, stratum radiatum of CA3 and all of stratum oriens at 8 years, but not the stratum radiatum of CA1, pyramidal cell layer of all subregions and the hilus. Even at the age of 11, myelinization was not complete in the hilus. An adult-like pattern of calbindin immunoreactivity can be observed at 11 years of age.

All of these data show periods when growth is taking place and is completed, but do not reveal when the networks are functional, which may occur somewhere within these periods. Even if a particular network arrives at maturity in the absence of vestibular inputs, the hippocampus is a highly plastic structure and would be expected to integrate substitutive inputs easily. However, the hippocampal atrophy in adult neurectomy patients would suggest that the absence of vestibular input in childhood would also impair hippocampal development. This could have different impacts at the respective ages. The data presented above suggests that different types of growth and maturation are occurring in the periods up to age 2–3, then leading up to 6–8, and then up to the age of 11 where adult-like characteristics appear.

## ONTOGENESIS OF SPATIAL NAVIGATION AND ORIENTATION IN CHILDREN

Several laboratories have examined the development of place navigation and related processes in young children, controlling and distinguishing from other simpler behaviors such as cued navigation. Lehnung et al. (1998) tested children in a 3.6-m diameter circular area closed off with curtains. Under dim lighting conditions, points on the floor were marked with lit fiberglass wires. The child had to first explore the group of points to find those selected as rewarded sites for that day, and then return and find them again. Both proximal cues on the floor (teddy bear, etc.) and wall cues were present. Various controls and experimental conditions were tested. While 5-year-olds employed the proximal cues, the 10-year-olds were able to use distal or proximal cues for orientation. Seven-year-olds were at a transition point, where half used only proximal cues, while the other half could use both cue types.

Overman et al. (1996) tested children in large real world environments, including a radial arm maze, a “dry Morris water maze” 0.9 m high and 3.6 m in diameter filled with plastic packing chips and a large 61 m circle in an outdoor playing field. In the radial arm maze, where each arm was rewarded only once per trial, children under 5 years old were impaired in both cued and non-cued versions when eight arms were used, showing spatial working memory performance inferior to older children and adults. (With only four arms open in the maze, these children succeeded at performing at adult levels however). When confronted with four forced choice trials, then, after a short delay, they were required to go to the remaining arms, the children under 5 years old performed at chance levels, 6- to 10-year-olds performed better, but only 20% of the latter achieved adult performance in this place learning task. In the dry Morris water maze, performance in finding the hidden “treasure chest” progressively improved among subjects until the age of 7 years. And only those children above the age of 8 years could localize the reward on a scale model of the maze. In the field the subjects were shown a goal location, blindfolded and driven along a circuitous route inside the circle, then asked to return to it. Performance improved in children 7 years and above, with 9-year-olds and older performing as well as adults. This is consistent with observations that 10-year-old children can resolve large-scale navigation tasks, but not 3- to 4-year-olds (Acredolo, 1976).

Virtual navigation tasks were developed as analogs of behavioral protocols used with rodents, and it is notable that many aspects of control of these tasks by spatial and non-spatial cues are similar across species. In cue competition experiments rats and humans display similar patterns of responses to removal of distal visual cues (e.g., Hamilton and Sutherland, 1999; Redhead and Hamilton, 2009). When the local apparatus is displaced in the same room after training both animal and human virtual navigation experiments suggest that distal cues control the directionality of navigation within the local apparatus (i.e., the pool). This too provides evidence for a fundamental similarity in how distal cues control navigation in the respective tasks. Thus it has been argued that parallel studies in humans and non-human animals could provide important information at multiple levels of analysis about the neurobehavioral relationships involved in place navigation and the development of these relationships. Interestingly, there are notable parallels in the development of spatial navigation abilities in rodents and humans in the respective tasks. Using a VMWT, Hoesing et al. (2000) found that children younger than age 7 did not reliably use a place navigation strategy to solve the VMWT but rather relied on various types of other strategies (e.g., circling a particular distance from the pool wall until the platform was encountered, randomly searching the pool). However, the pattern of successful performance by prepubertal children above age 7 (Hoesing et al., 2000) and post-pubertal adolescents (Hamilton et al., 2003) are comparable to that observed in adults (e.g., Hamilton et al., 2009) in that they learn to execute direct trajectories from multiple release points and persisted in searching at the target location during a probe trial with no escape platform.

Newcombe et al. (1998) found that from the age of 22 months, infants benefit from the use of the relations between distal cues to find a toy they had seen buried in a sand box. The children’s gaze at the site was interrupted and they started searching from a different point on the periphery. Ribordy et al. (2013) studied children aged from 2 to 5 years searching for rewards beneath an array of cups distributed in an open field arena 4 m × 4 m surrounded by opaque plastic walls on three sides. At 25–39 months of age, the infants could locate one rewarded cup out of the four presented (a simplified version of the task), albeit in the absence of local cues. However, 18- to 23-month-old infants were incapable of this. Thus both studies concur that near the age of 2 years capacities emerge for localization relative to distal cue configurations. Ribordy et al. (2013) point out that the age of 2 also marks the beginning of autobiographical memory as well as when the hippocampus reaches a certain state of maturity.

In summary, these studies suggest that there are at least two periods in development when new spatial skills appear. At the age of 2, infants are capable of rudimentary spatial localization (Ribordy et al., 2013), while capacity for place navigation emerges around 6–7 years of age in the Overman et al. (1996) and Hoesing et al. (2000) studies, and at the age of 11, adult performance appears. This is remarkably concordant with the three ages which mark milestones in hippocampal volume increase and myelination as noted in the previous section. Nevertheless, the interpretation of the coincidences of crucial ages in these developmental studies is clouded by the occurrence of other interrelated events at these ages. For example, at the age of 2, infants have recently gained mastery of independent walking and exploration, and this too might help elaborate spatial representations and promote hippocampal development.

Further advances could be made measuring performance in specific types of navigational processing using virtual environments such as the VMWT. Manipulations such as the combined cued navigation and pool translation in the VMWT permit to distinguish different ways of using distal visual cues for orientation alone or precise localization – processes that may be differentially affected by loss of vestibular function before or after key ages. Thus such approaches may prove useful in characterizing the effects of damage to the vestibular system on subsequent development of spatial navigation abilities. Importantly, because tasks of this type are administered via a computer program and interface experimental conditions can be controlled precisely, and can also be easily coupled with measures of functional brain activity (Cornwell et al., 2008), they will likely play an important role in further advancing our understanding of the behavioral consequences of early vestibular damage and their neurobiological bases.

## CRITICAL PERIODS

Rieser et al. (1986) compared sighted with blind adults who had lost vision early or late in life and had similar performance in evaluating perspective from an imaginary new observation point. When subjects walked without vision to the new point, pointing performance improved in the sighted and late-blinded subjects but not those of the early-blinded subjects. This suggests that early absence of vision leads to different types of representations of space. While there is a great variety in the performance levels of early blind vs late blind subjects, there seems to be a tendency for the former to employ route strategies while the latter engage mapping for navigation tasks (Thinus-Blanc and Gaunet, 1997).

The concept of critical period has been well developed for visual system ontogenesis (Imbert and Buisseret, 1975). Since multiple brain systems are respectively implicated in complementary orientation and navigational processing and they mature at different times, accurate vestibular signals at these times would be necessary for timely development. Critical periods for vestibular inputs would thus exist for each respective type of spatial processing.

## POSSIBLE MECHANISMS FOR VESTIBULAR DEFICITS LEADING TO COGNITIVE IMPAIRMENTS

Vestibular deficits could lead to diverse and distinct types of problems in cognitive processing problems with different respective underlying mechanisms. We showed (Wiener-Vacher et al., 2012b) that posturomotor control is delayed after a sudden complete vestibular loss due to meningitis before the age of independent walking. This led to long-lasting posturomotor instability in the absence of any neurological impairment. We suggested that the oscillopsia resulting from a complete lack of vestibular information in these children leads to dynamic and head–trunk instability. This could then contribute to secondary delays in learning processes (reading, writing, fine motor control) as well as building coherent representations of the body as well as its position relative to surrounding space. Much remains to be learned about the impact of complete or partial vestibular loss at different ages in children on the development posturomotor and fine motor control, oculomotor control in cognitive activities (reading, writing), spatial orientation, and body representation.

The absence of vestibular inputs to the hippocampus would lead to failure to establish normal brain representations of the body in space. A consequence of this would be difficulties in understanding spatial relations of environmental features. However, the resulting hippocampal atrophy could have a negative impact on other processes as well, like memory, context-dependent behaviors and relational reasoning. Another problem would derive from vestibular impairments leading to incomplete and imprecise sensorimotor feedback loops of many varieties. This would not be limited to activities involving head movements, since head immobility would be detected with lower certainty too. During development infants make myriad movements, and when the brain detects their outcomes, it can make corrections to refine sensorimotor coordination and build representations. Objects in three-dimensional space are understood not simply by their visual profile, but by how they feel, how they change appearance when manually rotated or when one walks around them, their weight, inertial and dynamic properties. Vestibular-impaired children’s problems with the gravity sense, the sense of orientation, awareness of the relations among one’s body parts and the distinction of self-movement from object movement could lead to impairments in their acquisition of knowledge through such sensorimotor feedback and interactive behavior. Another type of problem is related to absent or incomplete gravitational information which can lead not only to balance problems, but also to inaccurate compensation for gravitational forces on the body parts and environmental objects, particularly during movement. It has been demonstrated that the brain elaborates models of visually observed movement dynamics that distinguish those modulated by gravitational force (i.e., linear acceleration at 9.8 m/s^2^) from others (Zago and Lacquaniti, 2005). Other cognitive representations may also be built up from cerebral simulation of concordant sensorimotor loop activation experience, for example, mentally replaying walking through an environment could help build brain representations of that environment. Whether such experience is limited by choice (by a child who moves about and explores less frequently and less freely to avoid instable or disturbing situations) or by the incomplete nature of the sensory return information, this would nonetheless lead to poor spatial representations. Other sensory inputs can also be compromised in cases of vestibular deficits. For example, unstable gaze (in particular patients with spontaneous nystagmus) would impair visual perception and hence visual feedback from movements. Again the failure to distinguish visual field movements due to self or environmental features could have dramatic consequences. Many vestibular patients also suffer from partial or complete auditory deficits, which would impair access to echoes and ambient sound which also provide information about position and environmental structure.

## CONCLUSION

Our hypothesis is that an absence of vestibular information early in life can lead to reduced cognitive performance in several domains, as well as altered spatial cognitive representations (compared to children with no such vestibular deficit). This would persist a long time after vestibular compensation in the absence of appropriate therapy. The argument can be summarized as follows: the importance of the hippocampal system in spatial and other cognitive processing is supported by a vast experimental and neurological literature. Particularly striking evidence comes from neurophysiological recordings of place cell, HD cell and grid activity in rodents, activation of the hippocampus during virtual navigation in humans, and others. Theoretical arguments were advanced here for the roles of vestibular signals in building spatial reference frames and updating spatial representations. This is motivated by the observations that vestibular inactivation leads to the loss of HD and place cell activities as well as hippocampal atrophy and navigation deficits. Finally, the data on the ontogenesis of navigation behavior and hippocampal development converged remarkably on milestones at the ages of 2, 7, and 11. This leads to a refinement of our hypothesis wherein the onset of vestibular dysfunction prior to these milestones will delay the normal development of corresponding cognitive functions, and possibly lead to specific period-dependent changes in hippocampal structure and function. These may prove difficult to detect behaviorally because of rapid compensation of partial vestibular deficits, the high degree of plasticity that characterizes the hippocampal system, and variability among patients in their experiences learning to substitute other sensory modalities for the missing vestibular inputs. Nonetheless we predict that specific cognitive deficits will be detectable in at least a subpopulation of patients who lost vestibular function prior to the respective ages of 2, 7, and 11. One interesting question concerns the respective contributions of otolith and semicircular canal inputs for achieving these milestones. This knowledge would then lead to adapted therapies to help recover from these deficits.

In general, the issue of cognitive impact of deprivation of vestibular signal in children should have important consequences in patient care. Screening for vestibular loss should be done routinely in deaf children or in children with psychomotor developmental delays, who are often misdiagnosed as neurologically impaired or “slow.” Every effort should be made to avoid aggravating vestibular loss, for example, detect residual vestibular function prior to cochlear implantation in young patients and planning surgeries accordingly (Jacot et al., 2009). It is important to control for the possible effects of hearing impairment that are often associated with vestibular deficits. For example, vestibular deficits may impact on reading performance and further compromise language skills beyond impairments due to hearing loss. These screening tests must be comprehensive, including otolith testing which can now be performed easily, reliably, and relatively inexpensively via vestibular evoked myogenic potentials (VEMP; Jacot and Wiener-Vacher, 2008). While the caloric test remains a staple of the vestibulometry clinical battery, it is insensitive to otolith function, which we argue to be essential for establishing spatial reference frames.

## Conflict of Interest Statement

The author declares that the research was conducted in the absence of any commercial or financial relationships that could be construed as a potential conflict of interest.

## AUTHOR CONTRIBUTIONS

Each author wrote substantial portions of the text and all edited the entire manuscript.
